# Regional Variations in Esophageal Cancer Rates by Census Region in the United States, 1999–2008

**DOI:** 10.1371/journal.pone.0067913

**Published:** 2013-07-04

**Authors:** Jennifer Drahos, Manxia Wu, William F. Anderson, Katrina F. Trivers, Jessica King, Philip S. Rosenberg, Christie Eheman, Michael B. Cook

**Affiliations:** 1 Division of Cancer Epidemiology and Genetics, National Cancer Institute, National Institutes of Health, Department of Health and Human Services, Bethesda, Maryland, United States of America; 2 Cancer Prevention Fellowship Program, Division of Cancer Prevention, National Cancer Institute, National Institutes of Health, Bethesda, Maryland, United States of America; 3 Division of Cancer Prevention and Control, National Center for Chronic Disease Prevention and Health Promotion, Centers for Disease Control and Prevention, Atlanta, Georgia, United States of America; Peter MacCallum Cancer Centre, Australia

## Abstract

**Background:**

Assessment of cancer incidence trends within the U.S. have mostly relied upon Surveillance, Epidemiology, and End Results (SEER) data, with implicit inference that such is representative of the general population. However, many cancer policy decisions are based at a more granular level. To help inform such, analyses of regional cancer incidence data are needed. Leveraging the unique resource of National Program of Cancer Registries (NPCR)-SEER, we assessed whether regional rates and trends of esophageal cancer significantly deviated from national estimates.

**Methods:**

From NPCR-SEER, we extracted cancer case counts and populations for whites aged 45–84 years by calendar year, histology, sex, and census region for the period 1999–2008. We calculated age-standardized incidence rates (ASRs), annual percent changes (APCs), and male-to-female incidence rate ratios (IRRs).

**Results:**

This analysis included 65,823 esophageal adenocarcinomas and 27,094 esophageal squamous cell carcinomas diagnosed during 778 million person-years. We observed significant geographic variability in incidence rates and trends, especially for esophageal adenocarcinomas in males: ASRs were highest in the Northeast (17.7 per 100,000) and Midwest (18.1). Both were significantly higher than the national estimate (16.0). In addition, the Northeast APC was 62% higher than the national estimate (3.19% vs. 1.97%). Lastly, IRRs remained fairly constant across calendar time, despite changes in incidence rates.

**Conclusion:**

Significant regional variations in esophageal cancer incidence trends exist in the U.S. Stable IRRs may indicate the predominant factors affecting incidence rates are similar in men and women.

## Introduction

Although relatively uncommon in the United States (U.S.), with 17,460 incident cases estimated in 2012 [1], esophageal cancer is one of the most deadly malignancies with a 5-year survival rate of about 17% [2]. The two primary histological subtypes – esophageal adenocarcinoma (EA) and esophageal squamous carcinoma (ESCC) – account for 94% of all histologically-specified esophageal cancers [2]. Each histology is biologically distinct, with highly dissimilar risk profiles, differences in subsite localization, and stark differences with regard to worldwide incidence trends [3]. In the U.S., the incidence rate of EA has increased by 650% in white males over the last 35 years [4], [5]. In sharp contrast, the incidence of ESCC has steadily decreased. Although analyses of the National Cancer Institute's Surveillance, Epidemiology, and End Results (SEER) registries provide essential information on U.S. cancer trends, its ability to inform on regional trends is limited given that it includes only 28% of the U.S. population limited to specific geographic locales [6], [7].

Identifying regional patterns of esophageal cancer incidence may inform intervention strategies, afford the opportunity to elucidate causes for regional differences, and provide relevant information vital for making local cancer policy decisions. Population coverage across all census regions is necessary for such analyses and can be achieved when SEER registries are combined with registries that have participated in the Centers for Disease Control and Prevention's National Program of Cancer Registries (NPCR) [5], [8]. Leveraging the unique resources of NPCR and SEER, we assessed whether incidence rates and trends of EA and ESCC, as well as sex differences of these diseases, varied by geographic census region.

## Materials and Methods

### Data source

**Figure 1:**
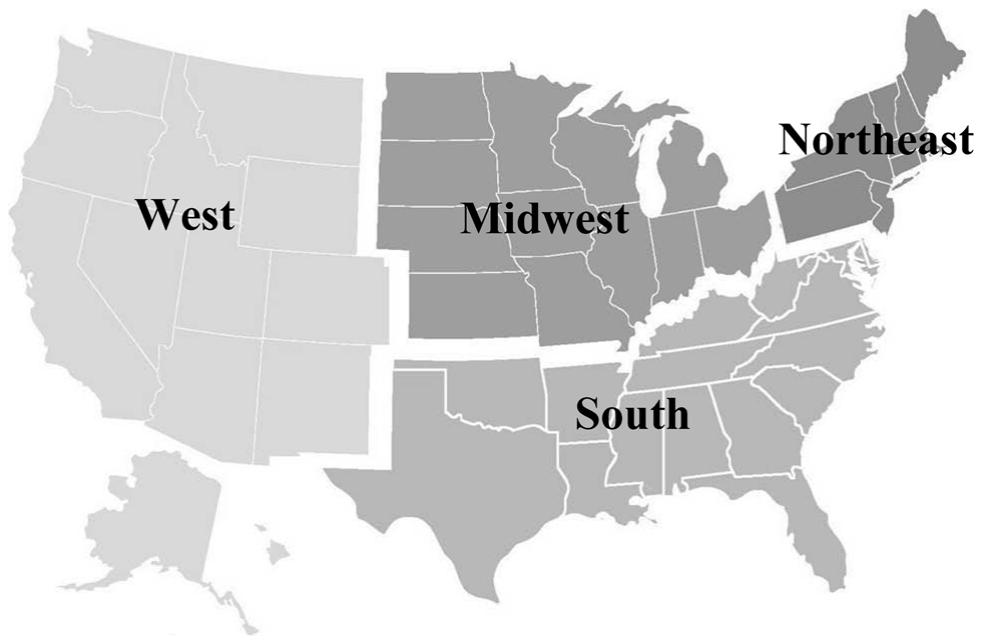
United States census regions. The states included in the Northeast, Midwest, South, and West census regions are highlighted. Coverage varied slightly by census region with nearly complete population coverage of the Northeast (100%), West (100%), and Midwest (98.8%), and slightly less population coverage in the South (76.0%).

Incident primary esophageal cancer cases were identified from population-based statewide cancer registries that participated in the NPCR or SEER programs [6], [8]. Each resource uses uniform methodologies for data collection and reporting [9]. When combined, the registries collect incidence data for the entire U.S. population. Data were continuously updated and monitored annually for quality. Data from statewide registries that met strict high-quality standards for all years (1999–2008) were included in the study [10]–[12]. Data in six states (AR, NC, MS, SD, TN, VA) and the District of Columbia did not meet case ascertainment and quality criteria for at least one of the years of the analytic period and were thus excluded. Data included from the remaining 44 states provided cancer registration coverage for 85.3% of the U.S. for the period 1999–2008. Given the number of esophageal cancer cases, after stratification by sex and histology, the geographic units were grouped using census region [13]. Figure 1 illustrates the states included in each of the four U.S. census regions. Coverage varied slightly by census region with nearly complete population coverage of the Northeast (100%), West (100%), and Midwest (98.8%), and less population coverage in the South (76.0%).

Esophageal cancer cases were classified by histologic subtype using morphology codes from the International Classification of Diseases for Oncology, Third Edition (ICD-O-3). EAs were defined as ICD-O-3: 8140–8575 and ESCC were defined as ICD-O-3: 8050–8084. Cases were restricted to whites aged 45–84 years of age because data for other races and ages were limited after stratifications by histology, sex, and census region. The variables captured and calculated were cancer count, person-years, and incidence rate per 100,000 age-adjusted to the 2000 US Standard Population (19 age groups – Census P25–1130). SEER*Stat software version 7.0.5 [14] was used to prepare counts of esophageal cancer and underlying populations by age and year of diagnosis for whites for the period 1999–2008.

### Statistical analyses

Age-standardized incidence rates (ASRs) were calculated for each calendar year and by histologic subtype (EA and ESCC), sex, and census region. Male-to-female incidence rate ratios (IRRs) were calculated by using the male ASR as the numerator and the female ASR as the denominator. Rates provided are per 100,000 person-years and 95% confidence intervals (CIs) were calculated for rates and ratios using the modification of Tiwari *et al*. [15]. Annual percent changes (APCs) were estimated by fitting a weighted-least-squares regression to the natural logarithm of the ASR for the period 1999–2008, with 95% CIs calculated using a normal approximation [16]. All data analyses were performed using SEER*Stat software version 7.0.5 [14] and SAS Software 9.2 [17].

Census region ASRs, APCs, and IRRs were compared with the national ASR, APC, and IRR and tested for statistically significant differences. To account for correlations due to overlapping regions, the method proposed by Tiwari *et al*. [18], [19] was used to test the difference of the ASRs, and the method proposed by Walters *et al*. [16] was used to test the difference of the APCs. A modified method proposed by Tiwari *et al*. that compares ratios and accounts for correlations due to the overlapping regions was used to test differences of the IRRs [20]. Differences between census regions and estimated national statistics were considered significant at P<0.05.

## Results

This analysis included 65,823 EA and 27,094 ESCC cases diagnosed in white adults aged 45–84 years during more than 778 million person-years for the calendar period 1999–2008. As shown in Table 1, the numbers of esophageal cancer cases were fairly evenly spread across census regions, with a slightly higher number of EA cases in the Midwest and a higher number of ESCC cases in the South, without adjustment for regional differences in age, sex and population size. Stark sex disparities were observed for both histological subtypes. The majority of EA and ESCC cases were diagnosed in men (86.5% and 64.0%, respectively). With regards to age, the largest proportion of EA cases and ESCC cases occurred in those 65–74 years.

**Table 1:** Esophageal cancer cases included for analysis: NPCR and SEER, whites, ages 45–84, 1999–2008.

	EA		ESCC	
	Count	%	Count	%
**National Total**	65,823	(70.8)*	27,094	(29.2)*
**Regional Counts**				
Northeast	16,137	(24.5)	6,936	(25.6)
Midwest	19,342	(29.4)	6,819	(25.2)
South	16,852	(25.6)	7,632	(28.2)
West	13,492	(20.5)	5,707	(21.1)
Total	65,823	(100.0)	27,094	(100.0)
**Sex**				
Male	56,965	(86.5)	17,339	(64.0)
Female	8,858	(13.5)	9,755	(36.0)
Total	65,823	(100.0)	27,094	(100.0)
**Age (years)**				
45–54	9,453	(14.4)	2,872	(10.6)
55–64	18,369	(27.9)	6,519	(24.1)
65–74	21,042	(32.0)	9,540	(35.2)
75–84	16,959	(25.8)	8,163	(30.1)
Total (45–84)	65,823	(100.0)	27,094	(100.0)
**Years**				
1999–2000	11,159	(17.0)	5,832	(21.5)
2001–2002	12,128	(18.4)	5,572	(20.6)
2003–2004	13,209	(20.1)	5,494	(20.3)
2005–2006	14,324	(21.8)	5,202	(19.2)
2007–2008	15,003	(22.8)	4,994	(18.4)
Total (1999–2008)	65,823	(100.0)	27,094	(100.0)

* Row percentages.

Abbreviations: EA, esophageal adenocarcinoma; ESCC, esophageal squamous cell carcinoma.

Estimates of national ASRs and APCs for the 10-year period are shown in Table 2. During 1999–2008, the overall ASRs for EA in men and women were 16.03 (95% CI: 15.90, 16.17) and 2.08 (95% CI: 2.04, 2.13) per 100,000 person-years, respectively. The incidence of EA increased in both sexes in the U.S. with APC estimates of 1.97% (95% CI: 1.67, 2.27) for men and 2.18% (95% CI: 1.44, 2.92) for women per year of calendar time. The national age-standardized incidence rate for ESCC was 4.93 (95% CI: 4.85, 5.00) per 100,000 in men and 2.30 (95% CI: 2.25, 2.34) per 100,000 in women. In contrast to EA, the incidence of ESCC in the U.S. decreased at −3.41% (95% CI: −3.91, −2.91) and −3.13% (95% CI: −3.80, −2.45) in men and women, respectively, per year of calendar time.

**Table 2:** Age-Standardized Incidence Rates and Annual Percent Changes Stratified by Histology, Sex, and Region in Whites, Aged 45–84, 1999–2008.

		Male		Female		Male-to-Female
EA		ASR∧	APC	ASR∧	APC	Rate Ratio
National		16.03 (15.90, 16.17)	1.97 (1.67, 2.27)	2.08 (2.04, 2.13)	2.18 (1.44, 2.92)	7.70 (7.52, 7.87)
Census Region	Northeast	**17.67** (**17.37, 17.97**)**	**3.19** (**2.58, 3.81)**	**2.51** (**2.41, 2.61**)**	**4.07** (**2.62, 5.54**)**	**7.04** (**6.74, 7.36**)
	Midwest	**18.09** (**17.82, 18.37**)**	**1.44** (**0.90, 1.98**)	**2.36** (**2.27, 2.45**)**	2.48 (1.11, 3.87)	7.68 (7.37, 8.01)
	South	**14.37** (**14.14, 14.60**)**	**2.59** (**2.01, 3.18**)	**1.70** (**1.63, 1.78**)**	2.08 (0.58, 3.06)	**8.43** (**8.05, 8.83**)
	West	**14.32** (**14.05, 14.58**)**	**0.80** (**0.15, 1.45**)	**2.08** (**0.58, 3.60**)**	**−0.52** (**−2.13, 1.12**)**	7.86 (7.47, 8.27)
**ESCC**						
National		4.93 (4.85, 5.00)	−3.41 (−3.91, −2.91)	2.30 (2.25, 2.34)	−3.13 (−3.80, −2.45)	2.14 (2.09, 2.20)
Census Region	Northeast	**5.81** (**5.64, 5.98**)**	−**2.26** (−**3.27,** −**1.25**)*	**2.52** (**2.42, 2.62**)**	−2.29 (−3.65, −0.91)	**2.31** (**2.20, 2.43**)
	Midwest	4.85 (4.70, 4.99)	−3.84 (−4.83, −2.85)	**2.17** (**2.08, 2.26**)**	−2.68 (−4.03, −1.31)	2.23 (2.12, 2.34)
	South	4.87 (4.73, 5.00)	−3.46 (−4.39, −2.53)	**2.16** (**2.08, 2.25**)**	−3.13 (−4.42, −1.83)	**2.25** (**2.14, 2.36**)
	West	**4.29** (**4.15, 4.44**)**	−3.71 (−4.83, −2.57)	**2.40** (**2.30, 2.50**)	−**4.41** (**−5.79,** −**3.02**)	**1.79** (**1.70, 1.89**)

Abbreviations: ASR, age-standardized incidence rate; APC, annual percentage change; EA, esophageal adenocarcinoma; ESCC, esophageal squamous cell.

∧ per 100,000 person-years.

**bold indicates significantly different than national rate** (**P≤0.05**), *P<0.01, **P<0.001.

Table 2 also displays these statistics by U.S. census region. As shown, for EA, there were significant regional variations in the ASR and APC. Compared with the national ASR of EA, the ASRs for the Northeast and Midwest were both significantly higher, and the South and West were both significantly lower, for both males and females. The APC for men in the Northeast was 62% higher than the national average (3.19% compared with 1.97%, P<0.001). Significantly lower APCs were observed for men in the Midwest and West, with the West having the lowest APC at 0.80% (0.15, 1.45). EA in females exhibited a similar pattern to males, with the highest APC occurring in the Northeast at 4.07% (2.62, 5.54) and lowest occurring in the West at −0.52% (−2.13, 1.12) and each significantly different than the national APC (P<0.001).

Some significant variations between regional and the national ASRs and APCs were also observed for ESCC (Table 2). Similar to patterns for EA in males, the male incidence rate of ESCC in the Northeast (5.81, 95% CI: 5.64, 5.98) was significantly higher (P<0.001) than the national average (4.93, 95% CI: 4.85, 5.00) and the male incidence rate of ESCC in the West (4.29, 95% CI: 4.15, 4.44) was significantly lower (P<0.001). Unlike EA, the APC of ESCC was decreasing during the 10-year period. Although ESCC was also decreasing in the Northeast, men in the Northeast had an APC (−2.26%, 95% CI: −3.27, −1.25) that was significantly less than the national trend (−3.41%, 95% CI: −3.91, −2.91, P<0.01). Women in the Northeast had a slightly, yet significantly, higher incidence rate (2.52, 95% CI: 2.42, 2.62) than the national average (2.30, 95% CI: 2.25, 2.34, P<0.001), although the difference in the APC in the Northeast compared with the national APC was not statistically significant.

The national IRR for EA was 7.70 (95% CI: 7.52, 7.87) (Table 2). Some regional EA IRRs did vary significantly from the national EA IRR–the Northeast had a significantly lower IRR (7.04, 95% CI: 6.74, 7.36) and South had a significantly higher IRR (8.43, 95% CI: 8.05, 8.83). The national ESCC IRR was 2.14 (95% CI: 2.09, 2.20). The ESCC IRR in the West (1.79, 95% CI: 1.70, 1.89) was significantly lower than the national IRR. Both the Northeast (2.31, 95% CI: 2.20, 2.43) and South (2.25, 95% CI: 2.14, 2.36) had higher IRRs than the national IRR for ESCC.

**Figure 2:**
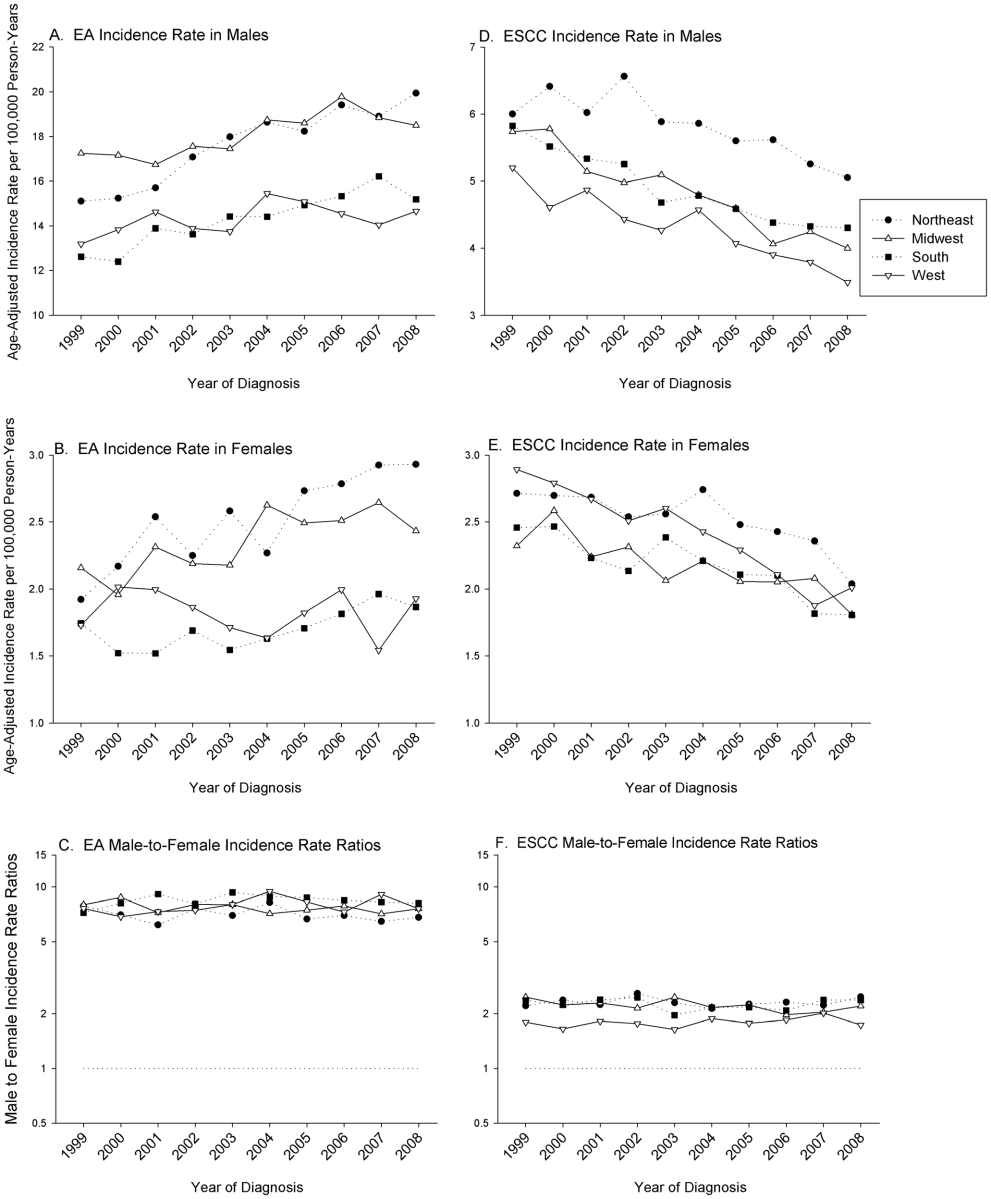
Age-adjusted incidence rates stratified by histology, sex, and region in whites, aged 45–84, 1999–2008. Panel A and B illustrate the age-adjusted incidence rate per 100,000 person-years by census region of the histologic subtype EA in men and women, respectively. Panel C shows the male-to-female incidence rate ratio (IRRs) of EA by calendar year. Panel D and E depicts the age-adjusted incidence rate of ESCC in men and women, respectively. Panel F shows the male-to-female incidence rate ratio (IRRs) of ESCC by calendar year. Rates graphed are per 100,000 person-years.

Figure 2 shows the age-adjusted incidence rates over calendar time stratified by histology, sex, and census region. Across all regions the incidence rate of EA has been steadily increasing, although regional variations are apparent. Consistent with the ASRs shown in Table 2, the male EA incidence rates in Northeast and Midwest census regions have been consistently higher than incidence rates in the South and West. Similar patterns, albeit with lower incidence rates, were observed for females in these census regions.

In contrast to EA rates across census region, ESCC has steadily decreased over the period analyzed (Figure 2). The Northeast region exhibited higher incidence rates in men at every year compared with the other three regions, which had similar rates and trends. For female ESCC cases, each region was decreasing similarly with little regional variation observed. The most recent incidence rates shown in Figure 2 for 2008, indicates that the Northeast had the highest rates of EA and ESCC in both sexes. The IRRs over calendar time displayed small fluctuations for both EA and ESCC, yet no pattern of change over time or between census regions were apparent.

## Discussion

In this analysis we observed significant geographic variability in EA and ESCC incidence rates by census region. The highest ASRs for EA were observed in the Northeast and Midwest for both men and women and were significantly higher than the national average (Table 2). In contrast, the South and West had significantly lower age-standardized rates than the national average. Our study demonstrates that national cancer estimates do not adequately address the current, and potentially future, esophageal cancer burden for a specific region.

Census regions differed by 3.77 per 100,000 in male EA, which is equivalent to ∼25% more cases per 100,000 in each of the Northeast and Midwest census regions relative to the West. The APC across census regions was also highly variable and has important implications. The APC for men in the Northeast was 3.19% (95% CI: 2.58, 3.81) in sharp contrast to 0.80% (95% CI: 0.15, 1.45) in the West. These data indicate a slight, but significant increase in EA for men in the West during the 10-year period. Although there are no clear explanations for these significant variations in EA incidence rates by census region, it is likely that key exposures important to this malignancy's pathogenesis in the U.S. population are causal to our observations. It is also possible that regional variation in EA is driven by a differential exposure to a strong, highly prevalent and yet unidentified causal factor as Edgren *et al*. hypothesized has led to the dramatic increase in EA incidence [21]. Thus further elucidation of our observations could help identify such a factor and aid preventative efforts across U.S. regions.

Our study also demonstrates that regional variations in ESCC rates and trends existed in 1999–2008. In men, the ASR for the Northeast indicated a higher cancer burden compared with other regions, and this was significantly different from the national estimate. In addition, the APC for Northeast men declined significantly less than the decline observed in the national average. Also mirroring the trends of EA in men, the ASR for ESCC in the West was the lowest of all census regions and significantly less than the national average.

It is not clear why the Northeast has had a generally higher esophageal cancer burden for both histologic subtypes relative to the other census regions assessed, or why the West consistently has a lower burden of each of these histologies, especially given that EA and ESCC are distinct in their risk factors. To supplement our findings we explored regional prevalence of exposure to major risk factors of these malignancies extracted from the 2007 NHIS survey data (Table S1). The prevalence of obesity was higher in the Northeast and Midwest, which may lead to increased risk of EA in those census regions. Regional variations in gastroesophageal reflux and cigarette smoking were inconsistent with our observed EA incidence trends by census region. Regional variations in alcohol consumption and cigarette smoking, however, were consistent with ESCC trends observed in our analysis. The Northeast had the highest prevalence of moderate or heavy alcohol consumption and above average ever smoking in both sexes. The higher regional exposure to smoking and drinking may partly explain why rates of ESCC are not declining as rapidly in the Northeast as other regions. Although data is somewhat suggestive, it does not clearly support significant differences in risk factor prevalence by census region in the year evaluated. Furthermore geographic variation in exposure to risk factors and trends in prevalence would be better explored in data which can also take into account the latency period, often estimated to be prolonged, between exposure and diagnosis of disease. This should be a focus of future studies.

Male predominance is a striking feature of esophageal cancer and, as reported in many other studies, we observed a very high IRR for EA (7.70, 95% CI: 7.52, 7.87) and a moderately high IRR for ESCC (2.14, 95% CI: 2.09, 2.20). Interestingly the IRR for each histotype remained fairly constant across the decade, even as the incidence rates were changing. This suggests that the underlying etiology of the sex disparity is unrelated to the factors that are driving increases in the incidence rate of EA and decreases in the incidence of ESCC. It also suggests that the predominant factors affecting changes in incidence rates over calendar time affect each sex in a relatively similar manner.

This study provides updated national and novel regional estimates of esophageal cancer incidence and trends by histology and sex. Our findings extend the timeframe analyzed in previous publications and demonstrate that ESCC incidence rates have continued to fall through 2008 [22]–[26]. We also observed that EA rates in the U.S. continued to increase with an APC of 1.97% (95% CI: 1.67, 2.27). Geographic differences in esophageal cancer incidence have been previously reported, however such studies relied on data with poor geographic distribution and focused on rates in individual cancer registries [3], [27]. The focus of our analysis was to assess sex and regional differences which builds on the foundation set by Trivers *et al* in her analysis of 1998–2003 data in NPCR and SEER that reported incidence rates and trends in esophageal cancer by census region [28]. The extended period and stratification by sex provides the ability to assess our hypotheses using sex specific overall rates as well as good statistical power to detect changes over calendar time.

There are several limitations of our study. NPCR and SEER registries are important and rich data sources for examining cancer incidence rates and trends, however, risk factor information is not available, so we were unable to directly investigate the influence of known risk factors. In order to provide stable estimates, our analyses were restricted to whites and, therefore, are not generalizable to other races. Cancer registry coverage across census regions was not uniform and it is possible that the missing population could differ from the covered population. Lastly, given our stratification by histology and sex, data were too sparse to analyze by census division or state level. To examine differences in rates and trends by census division or state, future studies could limit the analyses to men or combine data for each sex.

Our analysis has several notable strengths. Our study is the first to examine incidence rates and trends over time for esophageal cancer by census region. Despite the variation in coverage, the information provided by the NPCR-SEER data has far greater population coverage than SEER alone (85% versus 28% after exclusions). The analysis relied on data from statewide registries that met strict high-quality standards and encompassed a large proportion of the U.S. population, thus allowing for precise estimates of incidence rates by calendar year and age-specific incidence rates during the decade investigated.

Our comprehensive analysis demonstrates significant regional variations in the incidence and annual percent change in incidence rates of EA and ESCC among whites in the U.S. It will be interesting to see whether future studies of EA can identify risk factors that drive some of the regional heterogeneity of this malignancy, including the higher incidence rates in the Northeast and Midwest. We hypothesize here that the trends observed for ESCC may be influenced by regional exposure to risk factors, since we observed cigarette smoking and alcohol consumption patterns that roughly underlie the observed incidence rates of this malignancy. Novel study designs that can test these hypotheses further are needed. Finally, the stability of the IRRs for both histologic types is indicative that the causes of sex disparities in these diseases are potentially distinct from the extensive regional differences in incidence observed. Our findings underscore the importance of examining geographic and sex disparities in esophageal cancer and suggest that national cancer estimates may not adequately reflect specific geographic locales.

## Supporting Information

Table S1 NHIS 2007: Prevalence of Major Risk Factors of EA and ESCC. Our supplemental data relied on the National Health Interview Surveys (NHIS), which conducts nationally representative surveys of the health of civilian, non-institutionalized U.S. population. Using data from the 2007 NHIS, we calculated census region prevalence of reflux, obesity, ever-smoked cigarettes, and moderate to heavy alcohol consumption among whites aged 45–84 years, stratified by sex and census region.
(DOC)

## References

[1] American Cancer Society (2012) Cancer Facts and Figures. Atlanta: American Cancer Society.

[2] Surveillance, Epidemiology, and End Results (SEER) Program. SEER*Stat Database: Incidence – SEER 18 Regs Research National Cancer Institute, DCCPS, Surveillance Research Program, Surveillance Systems Branch, released April 2012, based on the November 2011 submission. Avaiable: http://www.seer.cancer.gov.

[3] Bollschweiler E, Wolfgarten E, Gutschow C, Holscher AH (2001) Demographic variations in the rising incidence of esophageal adenocarcinoma in white males. Cancer 92: 549–555. 11505399 10.1002/1097-0142(20010801)92:3<549::aid-cncr1354>3.0.co;2-l

[4] Cook MB, Chow WH, Devesa SS (2009) Oesophageal cancer incidence in the United States by race, sex, and histologic type, 1977–2005. Br J Cancer 101: 855–859. 19672254 10.1038/sj.bjc.6605246 PMC2736840

[5] Surveillance Epidemiology and End Results (SEER) Program. SEER*Stat Database: Incidence–SEER 9 Regs Research Data, Nov 2009 Sub (1973–2007). Linked To County Attributes–Total US, 1969–2007 Counties, National Cancer Institute, DCCPS, Surveillance Research Program, Cancer Statistics Branch, released April 2010, based on the November 2009 submission. Available: http://www.seer.cancer.

[6] Hankey BF, Ries LA, Edwards BK (1999) The surveillance, epidemiology, and end results program: a national resource. Cancer Epidemiol Biomarkers Prev 8: 1117–1121. 10613347

[7] Number of Persons by Race and Hispanic Ethnicity for SEER Participants (2000) Census Data. Surveillance Epidemiology and End Results Program.

[8] Centers for Disease Control and Prevention, Division of Cancer Prevention and Control, National Program of Cancer Registries: United States Cancer Statistics (USCS) Publication Standards.

[9] Wingo PA, Jamison PM, Hiatt RA, Weir HK, Gargiullo PM, et al (2003) Building the infrastructure for nationwide cancer surveillance and control–a comparison between the National Program of Cancer Registries (NPCR) and the Surveillance, Epidemiology, and End Results (SEER) Program (United States). Cancer Causes Control 14: 175–193. 12749723 10.1023/a:1023002322935

[10] Zippin C, Lum D, Hankey BF (1995) Completeness of hospital cancer case reporting from the SEER Program of the National Cancer Institute. Cancer 76: 2343–2350. 8635041 10.1002/1097-0142(19951201)76:11<2343::aid-cncr2820761124>3.0.co;2-#

[11] Thoburn KK, German RR, Lewis M, Nichols PJ, Ahmed F, et al (2007) Case completeness and data accuracy in the Centers for Disease Control and Prevention's National Program of Cancer Registries. Cancer 109: 1607–1616. 17343277 10.1002/cncr.22566

[12] Centers for Disease Control and Prevention. National Program of Cancer Registries (NPCR). Technical Notes: USCS Publication Criteria. Centers for Disease Control and Prevention.

[13] Centers for Disease Control and Prevention. National Program of Cancer Registries (NPCR). Technical Notes: Statistical Methods: Suppression of Rates and Counts. Centers for Disease Control and Prevention.

[14] Surveillance Research Program, National Cancer Institute SEER*Stat software Version 7.0.5. Available: http://www.seer.cancer.gov/seerstat.

[15] Tiwari RC, Clegg LX, Zou Z (2006) Efficient interval estimation for age-adjusted cancer rates. Stat Methods Med Res 15: 547–569. 17260923 10.1177/0962280206070621

[16] Walters KA, Li Y, Tiwari RC, Zou Z (2011) A Weighted-Least-Squares Estimation Approach to Comparing Trends in Age-Adjusted Cancer Rates Across Overlapping Regions. J Data Sci 8: 631–644. 22375146 PMC3286621

[17] SAS Institute Inc., SAS 9.2. Cary, NC: SAS Insitute Inc.

[18] Li Y, Tiwari RC, Zou Z (2008) An age-stratified poisson model for comparing trends in cancer rates across overlapping regions. Biom J 50: 608–619. 18615411 10.1002/bimj.200710430 PMC2536754

[19] Li Y, Tiwari RC (2008) Comparing trends in cancer rates across overlapping regions. Biometrics 64: 1280–1286. 18371122 10.1111/j.1541-0420.2008.01002.x PMC2588485

[20] Tiwari RC, Li Y, Zou Z (2010) Interval Estimation for Ratios of Correlated Age-Adjusted Rates. J Data Sci 8: 471–482. 22347884 PMC3279758

[21] Edgren G, Adami HO, Weiderpass E, Nyren O (2013) A global assessment of the oesophageal adenocarcinoma epidemic. Gut. 10.1136/gutjnl-2012-302412 22917659

[22] Brown LM, Devesa SS (2002) Epidemiologic trends in esophageal and gastric cancer in the United States. Surg Oncol Clin N Am 11: 235–256. 12424848 10.1016/s1055-3207(02)00002-9

[23] Younes M, Henson DE, Ertan A, Miller CC (2002) Incidence and survival trends of esophageal carcinoma in the United States: racial and gender differences by histological type. Scand J Gastroenterol 37: 1359–1365. 12523583 10.1080/003655202762671215

[24] Kubo A, Corley DA (2004) Marked multi-ethnic variation of esophageal and gastric cardia carcinomas within the United States. Am J Gastroenterol 99: 582–588. 15089886 10.1111/j.1572-0241.2004.04131.x

[25] Wu X, Chen VW, Ruiz B, Andrews P, Su LJ, et al (2006) Incidence of esophageal and gastric carcinomas among American Asians/Pacific Islanders, whites, and blacks: subsite and histology differences. Cancer 106: 683–692. 16388522 10.1002/cncr.21542

[26] Wu X, Chen VW, Andrews PA, Ruiz B, Correa P (2007) Incidence of esophageal and gastric cancers among Hispanics, non-Hispanic whites and non-Hispanic blacks in the United States: subsite and histology differences. Cancer Causes Control 18: 585–593. 17406989 10.1007/s10552-007-9000-1

[27] Kubo A, Corley DA (2002) Marked regional variation in adenocarcinomas of the esophagus and the gastric cardia in the United States. Cancer 95: 2096–2102. 12412162 10.1002/cncr.10940

[28] Trivers KF, Sabatino SA, Stewart SL (2008) Trends in esophageal cancer incidence by histology, United States, 1998–2003. Int J Cancer 123: 1422–1428. 18546259 10.1002/ijc.23691

