# Supplementary material for: Regional Variations in Esophageal Cancer Rates by Census Region in the United States, 1999–2008
Source: PLoS One. 2013 Jul 4;8(7):e67913. doi: 10.1371/journal.pone.0067913 (PMC3701616; doi:10.1371/journal.pone.0067913)
Supplement: Table S1 — NHIS 2007: Prevalence of Major Risk Factors of EA and ESCC. Our supplemental data relied on the National Health Interview Surveys (NHIS), which conducts nationally representative surveys of the health of civilian, non-institutionalized U.S. population. Using data from the 2007 NHIS, we calculated census region prevalence of reflux, obesity, ever-smoked cigarettes, and moderate to heavy alcohol consumption among whites aged 45–84 years, stratified by sex and census region. (DOC) [file pone.0067913.s001.doc]

|  | | | | | | | | |
| --- | --- | --- | --- | --- | --- | --- | --- | --- |
| **Table S1: NHIS 2007:  Prevalence of Major Risk Factors of EA and ESCC** | | | | | | | | |
|  |  | Ever Diagnosed with Reflux | Obese (BMI>=30) | Ever Smoked 100 Cigarettes | Moderate/Heavy Alcohol Intake | |  | |
|  | |
| Men | National | 19.6 (18.0, 21.2) | 30.6 (29.0, 32.3) | 57.5 (55.4, 60.0) | 27.7 (25.9, 29.4) | |  | |
|  | Northeast | 16.4 (12.4, 20.3) | 31.7 (27.0, 36.4) | 57.1 (53.1, 61.2) | 31.9 (28.9, 34.9) | |  | |
|  | Midwest | 21.0 (17.7, 24.2) | 33.1 (29.7, 36.5) | 56.5 (52.1, 60.9) | 27.7 (24.1, 31.2) | |  | |
|  | South | 19.7 (17.0, 22.3) | 30.5 (27.8, 33.2) | 59.2 (56.0, 62.3) | 24.5 (21.2, 27.7) | |  | |
|  | West | 20.3 (17.0, 23.5) | 27.0 (24.2, 29.9) | 56.5 (51.3, 61.7) | 28.4 (24.4, 32.4) | |  | |
| Women | National | 17.3 (15.9, 18.7) | 33.1 (31.4, 34.7) | 42.3 (40.6, 44.0) | 13.0 (11.8, 14.1) | |  | |
|  | Northeast | 18.4 (14.2, 22.5) | 33.6 (30.7, 36.5) | 47.2 (44.1, 50.4) | 16.1 (12.8, 19.4) | |  | |
|  | Midwest | 16.7 (14.4, 19.0) | 34.0 (29.9, 38.0) | 43.1 (39.1, 47.2) | 10.0 (8.1, 11.9) | |  | |
|  | South | 19.2 (16.6, 21.9) | 31.8 (29.3, 34.4) | 40.7 (38.1, 43.2) | 11.6 (9.9, 13.3) | |  | |
|  | West | 14.1 (12.1, 16.1) | 33.5 (30.5, 36.5) | 39.9 (36.0, 43.8) | 15.9 (12.8, 18.9) | |  | |
|  |  |  |  |  | |  | |  |
|  | Prevalence %., (95% Confidence Interval) | | |  | |  | | |
